# CO_2_-Philic Thin Film Composite Membranes: Synthesis and Characterization of PAN-*r*-PEGMA Copolymer

**DOI:** 10.3390/polym9070219

**Published:** 2017-07-06

**Authors:** Madhavan Karunakaran, Mahendra Kumar, Rahul Shevate, Faheem Hassan Akhtar, Klaus-Viktor Peinemann

**Affiliations:** Advanced Membranes and Porous Materials Center, 4700 King Abdullah University of Science and Technology (KAUST), Thuwal 23955-6900, Saudi Arabia; madhavan.karunakaran@kaust.edu.sa (M.K.); rahul.shevate@kaust.edu.sa (R.S.); faheem.akhtar@kaust.edu.sa (F.H.A.); klausviktor.peinemann@kaust.edu.sa (K.-V.P.)

**Keywords:** polyacrylonitrile, polyethylene glycol, copolymer, composite membranes, gas separation

## Abstract

In this work, we report the successful fabrication of CO_2_-philic polymer composite membranes using a polyacrylonitrile-*r*-poly(ethylene glycol) methyl ether methacrylate (PAN-*r*-PEGMA) copolymer. The series of PAN-*r*-PEGMA copolymers with various amounts of PEG content was synthesized by free radical polymerization in presence of AIBN initiator and the obtained copolymers were used for the fabrication of composite membranes. The synthesized copolymers show high molecular weights in the range of 44–56 kDa. We were able to fabricate thin film composite (TFC) membranes by dip coating procedure using PAN-*r*-PEGMA copolymers and the porous PAN support membrane. Scanning electron microscopy (SEM) and atomic force microscopy (AFM) were applied to analyze the surface morphology of the composite membranes. The microscopy analysis reveals the formation of the defect free skin selective layer of PAN-*r*-PEGMA copolymer over the porous PAN support membrane. Selective layer thickness of the composite membranes was in the range of 1.32–1.42 μm. The resulting composite membrane has CO_2_ a permeance of 1.37 × 10^−1^ m^3^/m^2^·h·bar and an ideal CO_2_/N_2_, selectivity of 65. The TFC membranes showed increasing ideal gas pair selectivities in the order CO_2_/N_2_ > CO_2_/CH_4_ > CO_2_/H_2_. In addition, the fabricated composite membranes were tested for long-term single gas permeation measurement and these membranes have remarkable stability, proving that they are good candidates for CO_2_ separation.

## 1. Introduction

Separation of carbon dioxide from light gases, such as methane, hydrogen and nitrogen has been widely applied in natural gas sweetening, post combustion (flue gas stream) and pre-combustion [1,2,3]. For CO_2_ separation membrane-based gas separation experienced significant growth in the past decades compared to the conventional separation processes (absorption, adsorption, and cryogenic distillation) because of its simplicity, low cost, high efficiency and low ecological footprint [4,5,6,7]. However, continuous improvement in membrane separation performance and cost reduction is further required to remain competitive. In terms of membrane separation performance polymer membranes suffer from a trade-off relationship, i.e., the higher permeability and the lower the selectivity [8]. For efficient gas separation membranes, it is important to have polymer membranes with high permeability as well as high selectivity. To be a competitive candidate for CO_2_ separation, it is envisioned that polymer membranes should have CO_2_ permeance of about 1000 GPU (Gas permeation unit, 1 GPU = 10^−6^ cm^3^ (STP)/(s·cm^2^·cmHg)) and an ideal CO_2_/N_2_ selectivity greater than 30 [9]. Various polymer membranes such as polysulfone [10], cellulose acetate [11], polyamides [12] polyimides [13] polyacetylenes [14] polycarbonates [15], poly(phenylene oxide) [16], poly(ethylene oxides) [17] and polyaniline [18] have been developed. However, only few of them have been explored for industrial application as commercial membranes due to their poor performance and scalability. Thus, the obstacles mentioned above need to be tackled for promoting commercialization of polymer membranes in industrial gas separation process.

In general, commercial polymer membranes are fabricated by two approaches: (i) anisotropic (asymmetric) membranes and (ii) thin film composite (TFC) membranes [6]. TFC membranes play an important role in industrial applications because of their easy scale up, low cost and high permeability. TFC membranes consist of a thin dense selective layer of 0.1–1 µm on the porous polymer support membrane thickness of 150–200 µm. The dense selective layer performs the molecular separation; it should be produced as thin as possible in order to obtain a high permeance. The microporous polymer support provides mechanical stability. In addition, the interface between the support and selective layer, the support layer porosity as well as pore size play the crucial role in the permeance and selectivity of the composite membranes. Polymeric gas separation membranes have also been manufactured by adding surface modifying macromolecules to the casting solution for asymmetric membrane preparation [19,20,21]. Recently, Membrane Technology Research, Inc. (MTR Inc., Menlo Park, CA, USA) has developed the hydrophilic TFC membrane (Polaris™) in a spiral wound model configuration, which is 10 times more permeable to CO_2_ than conventional cellulose acetate asymmetric membranes [22]. It has been noticed that poly ether based materials (PEG or PEO) are one class of polymer materials which have outstanding CO_2_ selectivity due to the strong affinity of the available polar ethylene oxide group towards CO_2_ [9,17].

The CO_2_-philic nature of PEG or PEO based polymer materials is mostly based on Lewis acid-base interaction between the ether oxygen (Lewis base) and the CO_2_ molecule (Lewis acid). However, the neat PEO has strong tendency to crystallize due to the helical structure of the chains that lead to the significant reduction in gas permeability [9]. To effectively suppress the crystalline characteristic of PEO chains, various strategies have been proposed by copolymerizing PEG monomers with hard segment, grafting or crosslinking PEG segments. Several PEO copolymer membranes such as poly(amide-*b*-ethylene oxide) (PA-*b*-PEO) [23], poly(butylene terephthalate-*b*-ethylene oxide) (PBT-*b*-PEO) [24], polycarbonate Z-*r*-poly(ethylene glycol) (PCZ-*r*-PEG) [25], poly(sulfone-*b*-ethylene oxide) (PSF-*b*-PEO) [26], poly(ethylene oxide)-*r*-poly(propylene oxide) (PEO-*r*-PPO-T6T6T) [27], poly(ethylene glycol) biphenyl ether methacrylate-*g*-poly(oxyethylene methacrylate) PEGBEM-*g*-POEM [28], poly(ethylene glycol)/poly(tetramethylene glycol) [29], poly(trimethylene terephthalate)-*b*-poly(ethylene oxide) (PTT-*b*-PEO) [30], and poly(2-[3,[3-(2H-benzotriazol-2-yl)-4-hydroxyphenyl] ethylmethacrylate)-*g*-poly(oxyethylenemethacryalate) (PBEM-*g*-POEM) [31] have been reported with high CO_2_ permeability and selectivity. The gas permeability was altered by controlling the hard and soft segments composition as well as the lengths of hard and soft segments. Very recently, Saito et al., developed amidoxime-functionalized PTMSP (AO-PTMSP) membranes for selective separation of CO_2_/N_2_ [32]. Among them, a commercially available block copolymer poly(amide-*b*-ethylene oxide) (PA-*b*-PEO, PEBAX) has been widely used in the fabrication of membranes for gas separation application [23,33,34,35,36,37].

PEBAX has the practical advantage that it is soluble in alcohols and it can be readily dissolved in ethanol-water mixture (7:3 *w/w*) at elevated temperature. TFC membranes can be produced using PEBAX solution by simple dip coating. Most high performance polymers reported are only soluble in aprotic solvents such as tetrahydrofuran (THF), *N*,*N*-dimethylformamide or *N*-methyl pyrrolidone, limiting the preparation of composite membranes, because the aprotic solvents could damage the porous polymer support membrane [24,26].

This motivated us to synthesize a copolymer that can be dissolved in alcohols or in ethanol/water mixture and can be used for the preparation of composite membranes by scalable dip coating method. We are interested in developing PEG copolymer based composite membranes with high CO_2_ permeability and selectivity. Herein, we report high performance CO_2_-philic TFC membranes using the synthesized polyacrylonitrile-*r*-methacrylate polyethylene glycol (PAN-*r*-PEGMA) copolymer. The series of PAN-*r*-PEGMA copolymers were synthesized via one-step free radical polymerization and the composite membranes were prepared on the porous PAN support membrane by dip coating. In this study, we discuss the synthesis of copolymers and their physical and the gas permeation properties.

## 2. Experimental Section

### 2.1. Chemicals and Materials

Poly(ethylene glycol) methyl ether methacrylate (PEGMA) with different molecular weight (*M_n_* = 300, 475 and 950 g/mol) and acrylonitrile (AN) were purchased from Sigma-Aldrich (Steinheim, Germany). PEGMA and AN monomers were passed through the prepacked column at 0.5 mL/min flow rate to remove hydroquinone and monomethyl ether hydroquinone inhibitors. 2,2-Azobis (2-methylpropionitrile) (AIBN, 98%) initiator was procured from Pfartz & Bauer (Waterbury, UK), which was purified through recrystallization from methanol. DMSO, DMF, cyclohexane, ethanol and deuterated dimethyl sulfoxide (DMSO-d^6^) were purchased from Sigma-Aldrich and used as such. PAN porous support membranes were purchased from GMT Membrantechnik, Rheinfelden, Germany.

### 2.2. Synthesis of Polyacrylonitrile-r-Polyethylene Glycol Methacrylate (PAN-r-PEGMA) Copolymer

PAN-*r*-PEGMA copolymers were synthesized by free radical polymerization reaction using AIBN initiator at elevated temperature. The initial concentrations of PEGMA and acrylonitrile monomers are provided in Table 1. The typical procedure for synthesis of PAN-*r*-PEGMA copolymers was: AN and PEGMA were alternatively added into a round-bottom flask containing DMSO. To this AIBN (1 wt % to the total weight of the monomers) was added under stirring at RT and the flask along with resulting reaction mixture was then sealed using a rubber septum. Pure nitrogen gas was further purged slowly for 30 min to remove the dissolved oxygen from a reaction mixture. The reaction mixture was heated to 60 °C with constant stirring for 24 h. After 24 h the temperature was further raised to 80 °C for an additional 16 h to attain high molecular weight PAN-*r*-PEGMA copolymer. The solution mixture was cooled to RT and the resulting copolymer solution was precipitated in a 200 mL mixture of cyclohexane and ethanol (70:30 *v/v* %). The solvent mixture was decanted and 100 mL of ethanol was again poured to remove unreacted monomer and traces of initiator. The copolymer was left in ethanol under constant stirring for 24 h at RT and the copolymer was filtered and then dried in the vacuum oven at 40 °C for 12 h [38].

### 2.3. Composite Membrane Fabrication

About 3 wt % of PAN-*r*-PEGMA copolymer was dissolved in ethanol/water (70/30 *v/v* %) mixture at 80 °C for 8 h. TFC membranes were then produced by dipping the double side sealed porous PAN support membrane in the copolymer solution and dried at RT. The membranes were then vacuum dried at 50 °C for 24 h to remove residual solvent. To study an effect of coating thickness, we used copolymer solution concentrations of 1, 2 and 3 wt %. In all other studies 3 wt % of PAN-*r*-PEGMA copolymer was maintained for membrane fabrication.

### 2.4. Characterization

Fourier transform infrared (FTIR) spectra of the synthesized copolymers were recorded using a Nicolet iS10 spectrometer (Thermo Scientific Co., Waltham, MA, USA) in the frequency range of 4000–600 cm^−1^. The data were collected for 16 scans with a resolution of 4 cm^−1^ at RT and FTIR spectra were normalized. The ^1^H-NMR spectra were recorded on 600 MHz high-resolution FT-NMR spectrometer (Bruker, Rheinstetten, Germany). DMSO-d^6^ was used as the solvent and tetramethylsilane as the reference. The thermal behavior of the copolymers was analyzed using a thermal gravimetric analyzer (TGA Q5000, TA Instruments, New Castle, DE, USA). TGA data were recorded for the copolymers in the temperature range of 25–600 °C at the heating rate of 5 °C/min under nitrogen environment. DSC measurements (DSC Q2000, TA Instruments) were performed at a heating rate of 10 °C/min under nitrogen environment to determine the glass transition temperature (*T*_g_) of the synthesized copolymers. The samples were heated from 45–110 °C, and the scanned thermograms were used to determine the *T*_g_ value of the copolymers. Wide angle X-ray diffraction (WXRD) measurements were conducted on a Bruker D8 Advance X-ray diffractometer with Cu-Kα radiation (λ = 1.54 Å) operated at 40 kV and 40 mA. The 2-theta angular region between 3° and 50° was explored at a scan rate of 5°/min. The molecular weights of the copolymers were determined by Gel permeation chromatography (GPC; Agilent 1260, Richardson, TX, USA) equipped with infinity multi-detector using DMF solvent at 45 °C. Molecular weights of the copolymers were determined using the narrow molecular weight distribution poly(methyl methacrylate) standards. Morphologies of the membranes were observed on field emission scanning electron microscope (FESEM; FEI Quanta 200, FEI Co., Oregon, USA). Membrane morphologies were recorded at 5 kV acelerating potential and 10 mm working distance. The dried membrane samples were mounted on aluminum stubs using aluminum tape and gold sputtering was carrried out on the membranes before recording their SEM images. For cross-section morphology analysis TFC membranes were fractured in liquid nitrogen and their SEM images were recorded. The surface of the composite membranes was characterized by atomic force microscopy (AFM; Model 5400, Agilent) using a silicon nitride tip with a resonance frequency in the range of 76–263 kHz in a tapping mode.

### 2.5. Gas Permeation Measurements

Single gas permeation experiments were performed using a pressure increase test unit built at KAUST (constant volume, variable pressure). The effective area of TFC membranes was ~13 cm^2^. The permeability of gas through the composite membranes was measured at 25 °C using a pressure increase time-lag apparatus with a feed pressure of 500 mbar. The permeance value of TFC membrane was calculated using Equation (1):

(1) $$J=\frac{V.\;22.4}{R.\;T.\;A.\;t}\ln(\frac{p_{F}-p_{0}}{p_{F}-p_{P\;\left(t\right)}}\;)$$

where *V* is the permeate volume (L), *R* is the ideal gas constant (0.0831 bar·L·mol^−1^·K^−1^), *T* is the temperature (K), *A* is the membrane area (m^2^) and t is the time of measurement (s). $p_{F}$, $p_{0}$, and $p_{P\left(t\right)}$ (bar) are the pressures at the feed, permeate side at beginning, and permeate side at the end of the measurement, respectively. The membrane selectivity towards the gas was determined from ratio of the permeance for each component.

## 3. Results and Discussion

### 3.1. Synthesis of PAN-r-PEGMA Copolymers

**Scheme 1:**
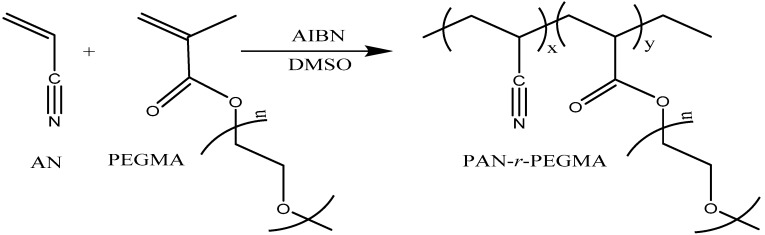
Reaction route for synthesis of PAN-*r*-PEGMA copolymers by free radical polymerization.

The series of PAN-*r*-PEGMA copolymers were synthesized via free radical polymerization at elevated temperature using AIBN (Scheme 1). The mole ratio of AN reacted with various molecular weights of PEG methacrylate is tabulated in Table 1. Initially the reaction was performed at 60 °C for 24 h to obtain high molecular weight PAN-*r*-PEGMA copolymers; the reaction was further carried out at 80 °C for an additional 16 h.

We synthesized PAN-*r*-PEGMA copolymers with different PEG compositions. We considered only the copolymers, which were soluble in ethanol/water mixture. The copolymers reported in Table 1 were soluble in ethanol/water mixture at 80 °C and formed defect free composite membranes. The weight average molecular weight (*M*_w_) and polydispersity index (PDI) of the synthesized copolymers obtained by GPC are given in Table 1. The M_w_ of the copolymers were in the range of 43 kDa–56 kDa with PDI of 1.96–3.6.

**Figure 1:**
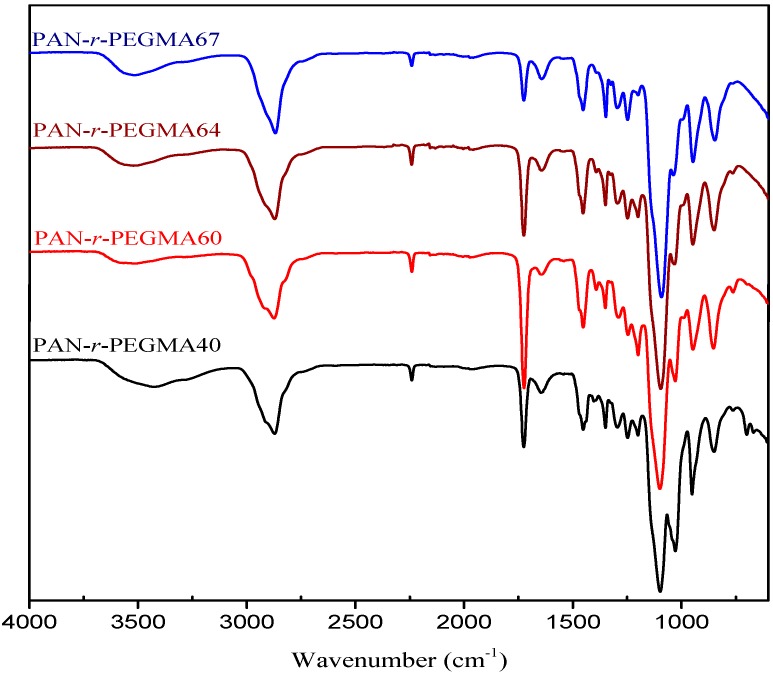
FTIR spectra for PAN-*r*-PEGMA copolymers.

PAN-*r*-PEGMA copolymers synthesized were analyzed by FTIR spectroscopy and Figure 1 shows FTIR spectra of PAN-*r*-PEGMA copolymers with varied amount of PEG content. The sharp absorption bands at 2245, 1257 and 1033 cm^−1^ are attributed to the stretching and bending vibrations of –C≡N of PAN segment. The strong band at 1081 cm^−1^ corresponds to the C–O–C stretching vibration of the PEG moiety, which indicates that PEGMA monomers were well copolymerized with AN. In addition, the intensity of the peak at 1081 cm^−1^ for C–O–C stretching increased in the order of PAN-*r*-PEGMA67 > PAN-*r*-PEGMA64 > PAN-*r*-PEGMA60 > PAN-*r*-PEGMA40, indicating an increase in PEG content of the copolymer. A sharp band observed at 1722 cm^−1^ is attributed to C=O stretching vibration of the acrylate ester group of PEGMA segment. The broad absorption bands at 2895 cm^−1^ correspond to –C–H stretching vibration of methylene groups present in the backbone of the copolymer and the side chain of PEGMA segment.

**Figure 2:**
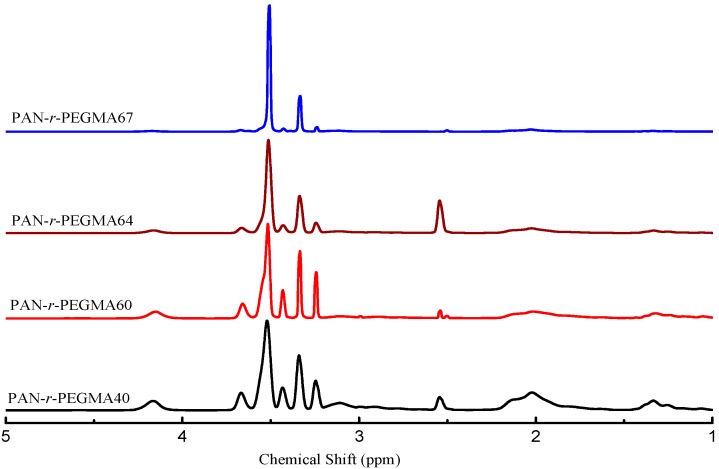
The ^1^H-NMR spectra for PAN-*r*-PEGMA copolymers in DMSO-d^6^ solvent.

In order to further confirm the formation of copolymers, all the polymers were investigated by NMR spectroscopy. The ^1^H-NMR spectra of the copolymers in DMSO-d^6^ solvent are illustrated in Figure 2.

The signals at *δ* = 2.02 and 3.23 ppm are assigned to –CH_2_– and –CH– protons of PAN segment. The characteristic peaks at δ = 4.16, 3.4–3.6, 2.11 and 1.32 ppm are due to –COO–CH_2_, –CH_2_–O–CH_2_–CH– and –CH_2_ protons of PEGMA segment in the copolymers. The peak of terminal –O–CH_3_ group in PEGMA segment appears at 3.10 ppm. NMR spectra confirm the copolymerization between AN and PEGMA in presence of AIBN at elevated temperature. These results confirm successful synthesis of PAN-*r*-PEGMA copolymers from AN and PEGMA with various molecular weights via free radical polymerization reaction.

**Figure 3:**
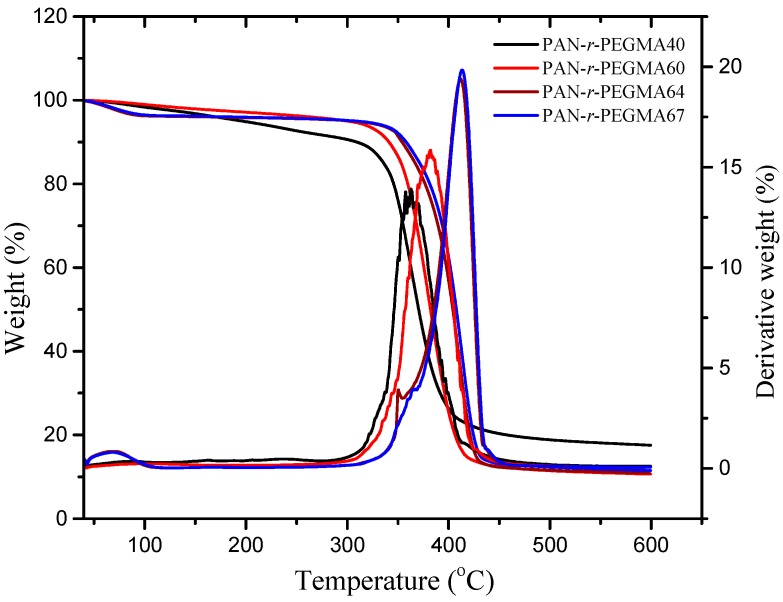
Thermal gravimetric analysis and derivative (DTG) curves for PAN-*r*-PEGMA copolymers.

**Figure 4:**
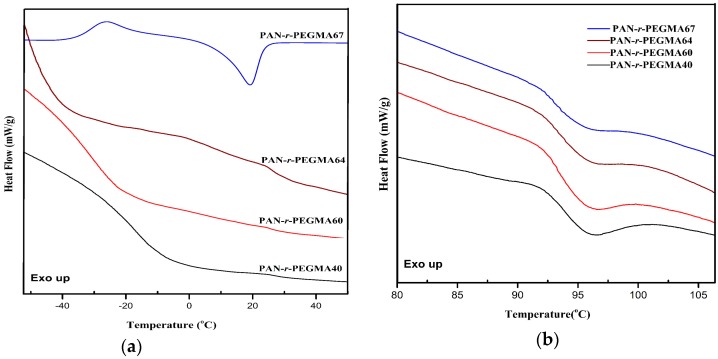
(**a**,**b**) The differential scanning calorimetry thermograms for PAN-*r*-PEGMA copolymers.

The thermal analysis of PAN-*r*-PEGMA copolymers were studied in the temperature range of 25–600 °C at the heating rate of 5 °C/min under nitrogen atmosphere. TGA thermograms of the copolymers are shown in Figure 3. The weight loss (%) for PAN-*r*-PEGMA67 copolymer was noticed at 100 °C; this could be ascribed to the presence of moisture in the synthesized copolymer. It can be visualized that single stage decomposition of the copolymers started above 320 °C. The thermal degradation temperature of the copolymers increased gradually with PEG content. The PAN-*r*-PEGMA64 and PAN-*r*-PEGMA67 copolymers were more thermally stable at least up to 356 °C in comparison to PAN-*r*-PEGMA40 and PAN-*r*-PEGMA60 copolymers. The increase in PEG content of the polymers usually decreases their thermal stability. However, here the thermal stability of the copolymers is enhanced with increase in PEG content. Overall, TGA results indicate good thermal stability of the synthesized copolymers, which is attractive for a potential gas separation material. The *T*_g_ values of the copolymers (see Table 1) were determined by DSC. The DSC thermograms for all the copolymers are presented in Figure 4a,b.

Two glass transition temperatures were obtained for the copolymers i.e., *T*_g1_ was observed in the range of −45 to −9 °C and *T*_g2_ was in the range of 95–97 °C. *T*_g1_ values in the range of −45 to −9 °C are related to the presence of pendant PEG side chains of the copolymer backbone. *T*_g1_ values declined on increase in PEG contents, this is ascribed to the increased mobility of the pendant PEG chains, which could enhance the total free volume of the copolymers. This factor is mainly responsible for improvement in gas transport properties, which enhance both solubility and diffusivity particularly for CO_2_ gas.

PAN-*r*-PEGMA40, PAN-*r*-PEGMA60, and PAN-*r*-PEGMA64 did not show any strong endothermic peaks, corresponding to the crystalline melting of PEG. Therefore, we conclude that all the copolymers were in the amorphous state. Among them PAN-*r*-PEGMA67 only showed a sharp melting point around 22 °C, indicating partial crystalline nature of the PEG side chain. This may be due to the high molecular weight (950 Da) of the PEGMA monomer compared to other PEGMA monomers used for the copolymer preparation. In general, *T*_gAB_ of the random copolymer (A-*r*-B) lies between *T*_gA_ and *T*_gB_. The *T*_gAB_ value depends on the ratio of A and B monomers used in the copolymer synthesis. In this study, the copolymers were synthesized from AN and PEGMA monomers while *T*_g_ values of the individual homopolymers are 95 °C (PAN) and −56 °C (PEGMA-300) [39,40]. We anticipated that *T*_g_ values of the random copolymers should be in between for above mentioned copolymers. However, *T*_g_ values for all of the copolymers were observed around 95 °C (Figure 4b), which is indicating that the *T*_g_ belongs to the segmental mobility of the main chain of PAN backbone. The PAN-*r*-PEGMA40 (low PEG content) copolymer showed a *T*_g_ at 97 °C and for the PAN-*r*-PEGMA67 copolymer (high PEG content) at 95 °C. A slight shift in the *T*_g_ value in the range of 97–95 °C was observed with increase in amount of PEG of the copolymers. As we mentioned earlier, the crystalline nature of PEG molecules affects the gas transport properties.

**Figure 5:**
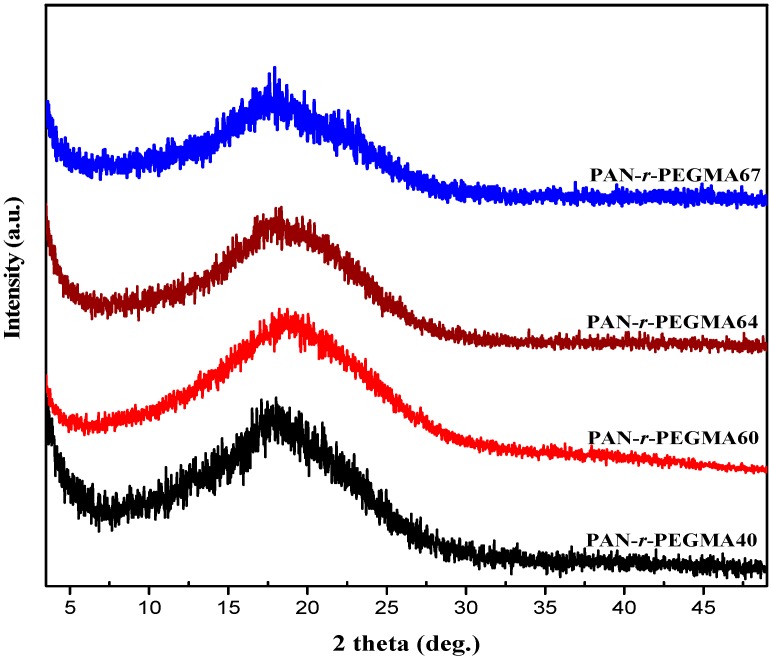
The WXRD patterns for PAN-*r*-PEGMA copolymers.

Wide angle XRD (WXRD) was employed to analyze the crystalline behavior of the copolymers. Figure 5 shows WXRD patterns of the PAN-*r*-PEGMA copolymers in which the intensity of X-ray scattering is plotted against the diffraction angle 2θ. The broad 2θ peaks were observed near 18° and sharp crystalline peaks are absent in the WXRD patterns of all copolymers. This confirms the amorphous nature of the synthesized copolymers.

### 3.2. Membrane Preparation and Morphology

Thin film composite membranes were successful fabricated on the porous PAN support membranes from a copolymer dissolved in ethanol/water (70/30 *v/v* %) mixture by dip coating method.

**Figure 6:**
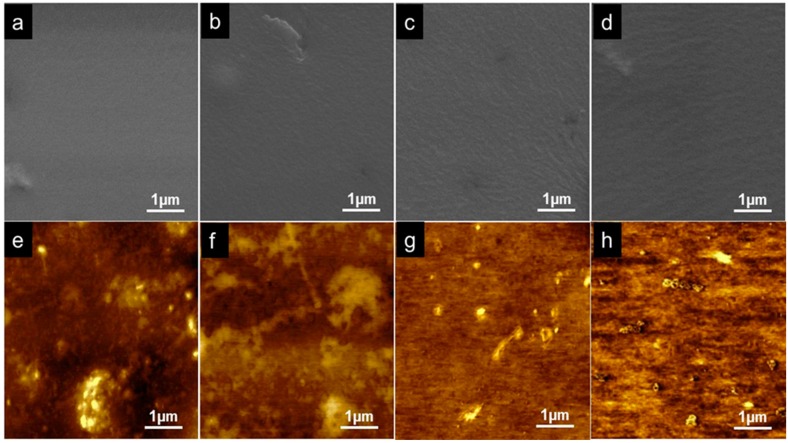
The surface SEM and AFM images of PAN/PAN-*r*-PEGMA composite membranes (**a**,**e**) PAN-*r*-PEGMA40; (**b**,**f**) PAN-*r*-PEGMA60; (**c**,**g**) PAN-*r*-PEGMA64, and (**d**,**h**) PAN-*r*-PEGMA67.

**Figure 7:**
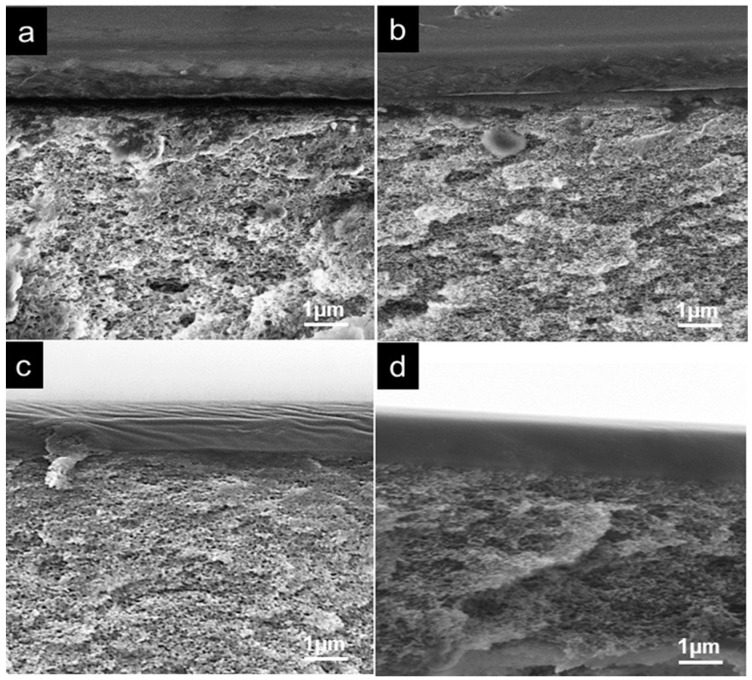
The cross-section SEM images of the composite membrane (**a**) PAN-*r*-PEGMA40; (**b**) PAN-*r*-PEGMA60; (**c**) PAN-*r*-PEGMA64 and (**d**) PAN-*r*-PEGMA67.

We also made efforts to fabricate free-standing dense membranes using a copolymer solution by solvent evaporation at 50 °C for investigating their gas transport properties. After the evaporation of solvent, the obtained dense membranes were flexible but sticky in nature. Therefore, we were not able to evaluate gas transport properties of the free-standing membranes. The morphologies of the composite membranes were examined by scanning electron microscopy. All the membranes have smooth surface coating of PAN-*r*-PEGMA copolymers onto the top surface of porous PAN support membrane (Figure 6a–d). These images reveal the formation of a defect free selective layer of PAN-*r*-PEGMA copolymer onto the porous PAN support membrane through dip coating method. We anticipated that the microphase separation between PAN (relatively hydrophobic) and side chain PEG (hydrophilic) of the copolymers occurs in the microdomains of the membrane selective layer due to two glass transition temperatures of the copolymers. The microphase separation of the copolymers was investigated with AFM. The top surface layers of the composite membranes were imaged using AFM in a tapping mode. The microphase-separated morphology was observed for all the TFC membranes (Figure 6e–h). In order to see the adhesion of the copolymer selective layer with the porous PAN support layer, the membranes were fractured in liquid N_2_ and the cross-section images are depicted in Figure 7a–d.

Very thin and smooth coating layers are clearly visible on the top of the porous PAN support membrane. In this study, one of the copolymer segments is PAN and the porous support layer is also PAN. We expect therefore a good compatibility between support and coating leading to better gas separation properties. The thickness of membrane selective layers was measured from cross-section SEM images and the obtained values are tabulated in Table 2. The selective layer thickness of the composite membranes is in the range of 1.2–1.4 μm.

### 3.3. Gas Transport Properties of the Composite Membranes

**Figure 8:**
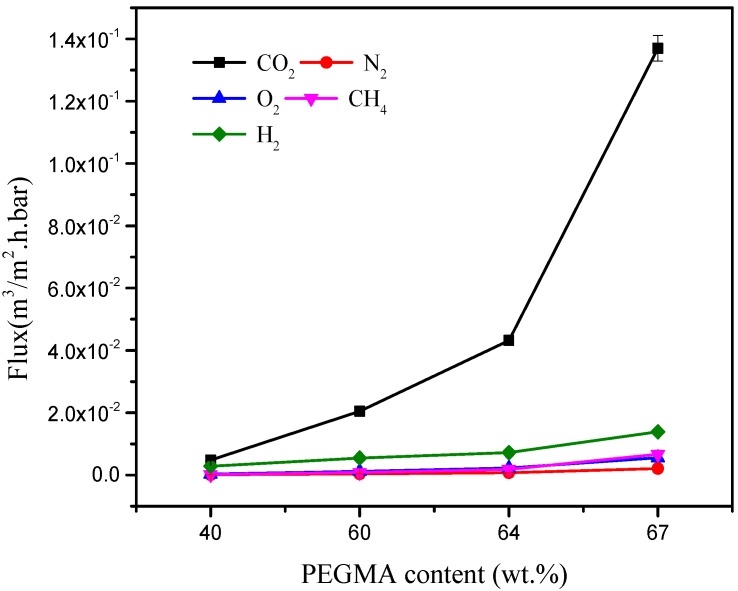
Plot of gas permeability against the content of PEGMA (wt %).

Pure gas permeation properties of the composite membranes were determined using N_2_, O_2_, H_2_, CH_4_, and CO_2_ gases at 25 °C. Figure 8 shows the gas permeability data for composite membranes fabricated from the series of PAN-*r*-PEGMA copolymers.

The order of gas permeability of the composite membranes at 25 °C is CO_2_ > H_2_ > CH_4_ > O_2_ > N_2_, which is in good agreement with the reported data in the literature for PEG based polymers [23,24,28,30,33,35]. All the composite membranes show the same gas permeability trend (CO_2_ > H_2_ > CH_4_ > O_2_ > N_2_) and the gas permeability of the composite membranes increases with increase in the content of PEG. The CO_2_ permeability of composite membranes increases strongly with content of PEGMA monomer in the initial synthesis composition. This trend is similar to the other reported PEO block copolymer composite membranes [24,25,26,27,28,29]. It is reported that the performance of the membranes based on PEO polymers is affected by the amount and molecular weight of PEO. The PAN-*r*-PEGMA derived composite membranes have good gas permeabilities. In particular, the PAN-*r*-PEGMA67 copolymer composite membrane has high gas permeability for all gases especially for CO_2_. The CO_2_ gas flux of about 1.37 × 10^−1^ m^3^/m^2^·h·bar was obtained for the PAN-*r*-PEGMA67 composite membrane.

In order to position PAN-*r*-PEGMA composite membrane in the PEG based polymer membrane library, we compared the gas permeability of commercial PEBAX1657 (PEO content 60%) with PAN-*r*-PEGMA composite membranes. The PEBAX1657 composite membranes were prepared on the porous PAN support membrane by dip coating using 3 wt % of PEBAX1657 solution in ethanol/water (70/30 *v/v* %) mixture. The CO_2_ flux of the PEBAX composite membrane was 1.25 × 10^−1^ m^3^/m^2^·h·bar. These flux values reveal that the CO_2_ flux and the order of gas permeability (CO_2_ > H_2_ > CH_4_ > O_2_ > N_2_) for the PAN-*r*-PEGMA67 composite membranes are similar to the PEBAX1657 composite membrane. However, the CO_2_ gas permeability of the PAN-*r*-PEGMA40 composite membrane is 10 times lower than PEBAX1657 composite membrane permeability, which is due to low content of PEG (40%). These results are clearly demonstrating the improvement in CO_2_ gas permeability of the composite membranes through the ethylene oxide units of PEG segments. To calculate the membrane permeability coefficient, the thickness of the composite membranes obtained from SEM images were taken into account and calculated using the following steady-state permeability of gas equation:

(2) $$P_{A}=\;\frac{N_{A}\;l}{p_{2A}-p_{1A}}$$

where $N_{A}$ is the steady state flux of gas through the membrane (cm^3^ (STP)/cm^2^ s), l is the membrane thickness (cm), and $\;p_{2A}$,$\;p_{1A}$, are the upstream and downstream pressures (cm Hg). The selective layer thickness of PAN-*r*-PEGMA composite membranes and their permeability data are included in Table 2. The ideal selectivity of the membrane for gas A over gas B is the ratio of their pure gas permeability $P_{\;A}$ and $P_{B}$ as given in Equation (3):

(3) $$\propto_{\frac{A}{B}}=\;\frac{P_{A}}{P_{B}}$$

The gas pair selectivity was calculated by using Equation (3) and the obtained values are also tabulated in Table 2. The composite membranes exhibit high selectivity for the CO_2_/N_2_ gas pair*.* (Table 2). The CO_2_/N_2_ selectivies of the composite membranes increased with increase in the content of PEGMA. The CO_2_/N_2_ selectivity of the PAN-*r*-PEGMA40 composite membrane is ~52 wheras the CO_2_/N_2_ selectivity of the PAN-*r*-PEGMA67 composite membrane is 65 due to the high content of PEGMA (950 Da) in the copolymer. The CO_2_/N_2_ selectivies of PAN-*r*-PEGMA compsite membranes are higher than the selectivities of the membranes made from comercial PEBAX1657 (Table 2). Interestingly, the composite membrane with low content of PEGMA has better CO_2_/CH_4_ selectivity and the selectivity decreased with increase in content of PEGMA. In comparison to PEBAX1657 composite membrane, CO_2_/N_2_, CO_2_/CH_4_, and CO_2_/H_2_ selectivies of the PAN-*r*-PEGMA composite membranes are high. This reveals that PAN-*r*-PEGMA composite membranes are more CO_2_-philic.

**Figure 9:**
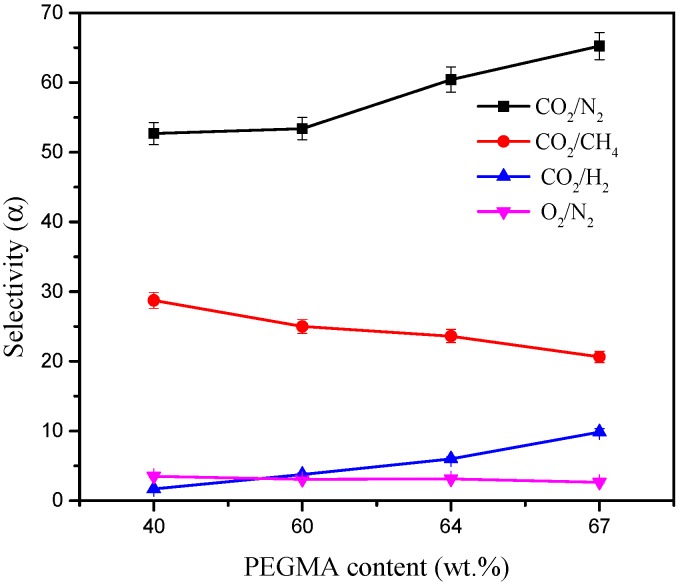
Plot of membrane gas selectivities versus content of PEGMA (wt %).

Figure 9 shows an effect of PEGMA content on gas selectivies of the composite membranes. It is observed that PAN-*r*-PEGMA composite membranes have resonable O_2_/N_2_ gas selectivies, which are in the range of 2.3–3.5. From the Figure 9, it was observed that the selectivies of CO_2_/CH_4_ and O_2_/N_2_ decreased with increase in the content of PEGMA. However, the CO_2_/N_2_ and CO_2_/H_2_ selectivies increased with PEGMA content. To study an effect of the selective layer thickness, we chose the PAN-*r*-PEGMA67 copolymer and varied the coating solution thickness by dip coating using different copolymer concentrations (1 to 3 wt %) in ethanol/water mixture.

**Figure 10:**
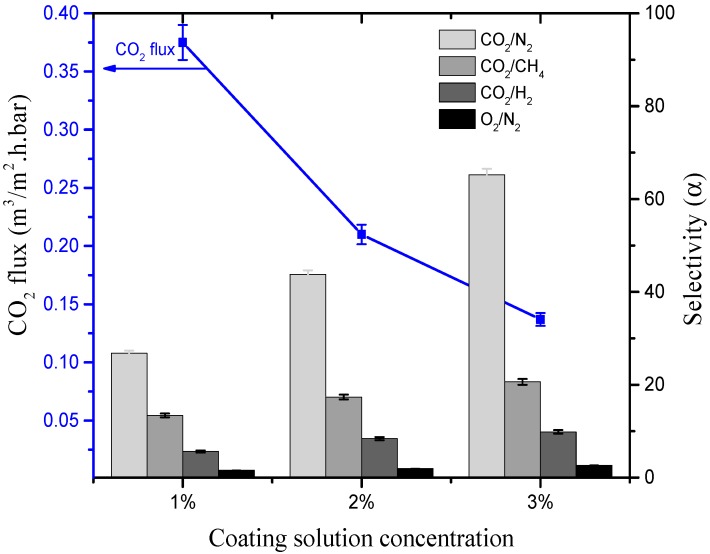
Effect of CO_2_ flux and gas selectivities at various concentration of PAN-*r*-PEGMA67 copolymer coating solution.

Figure 10 shows the plot of different concentration of copolymer coating solution versus CO_2_ flux and gas pair selectivity. The selective layer thickness plays a crucial role for gas flux and selectivity of the composite membranes. We observe an increase in CO_2_ flux as the concentration of the copolymer coating solution decreases because of the reduced thickness of the selective layer. The CO_2_ permeability of the PAN-*r*-PEGMA67 composite membrane improves from 1.37 × 10^−1^–3.75 × 10^−1^ m^3^/m^2^·h·bar when 1 wt % copolymer solution was coated on the porous PAN support membrane. On the other hand, the selectivity of these membranes are extensively influenced by a thickness of copolymer coating layer. The CO_2_/N_2_ and CO_2_/CH_4_ selectivity decreased from 65–22 and from 20–13, respectively for the composite membranes prepared from 1 wt % PAN-*r*-PEGMA67 copolymer solution. This decrease in selectivity is due to the formation of pin holes in the top layer of the composite membranes at low concentration of PAN-*r*-PEGMA67 copolymer solution. PAN-*r*-PEGMA copolymers were flexible and soft and we examined, if the fabricated TFC membranes are stable for long-term gas separation application.

**Figure 11:**
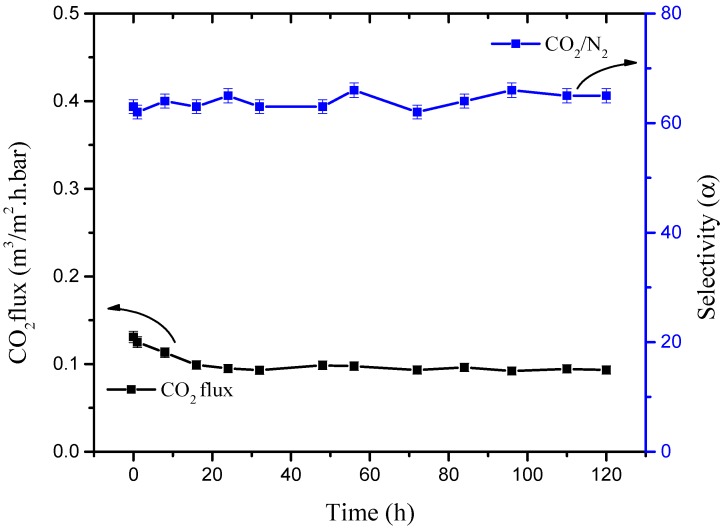
Long-term operation test for permeability of CO_2_ and N_2_ gas and the paired gas selectivity for PAN-*r*-PEGMA67 composite membrane. Gas permeation tests were conducted at 25 °C and 0.5 bar.

To test the long-term stability, the PAN-*r*-PEGMA67 composite membrane was tested in the time lag machine for 120 h. The membranes were kept under vacuum in the membrane test chamber throughout testing and the individual permeance of CO_2_ and N_2_ gases was measured at various time intervals for 120 h. The measured CO_2_ flux and CO_2_/N_2_ selectivity at various time intervals up to 120 h are illustrated in Figure 11. Figure 11 shows that an initial CO_2_ flux for PAN-*r*-PEGMA67 composite membrane was 1.37 × 10^−1^ m^3^/m^2^·h·bar and a slight decline in the CO_2_ flux (0.93 × 10^−1^ m^3^/m^2^·h·bar) was observed until 32 h and afterwards a stable flux with slightly reduction in CO_2_ flux was achieved up to 120 h. However, the CO_2_/N_2_ selectivity of the PAN-*r*-PEGMA67 composite membrane remained in the range of 63–66. These results prove that PAN-*r*-PEGMA composite membranes are stable for long-term separation application.

## 4. Conclusions

In summary, we have designed a new gas separation membrane material with enhanced gas transport properties. PAN-*r*-PEGMA copolymers were synthesized through a free radical polymerization at 80 °C in presence of AIBN initiator. The amount of PEG segments in the various copolymers was varied by altering the molar composition of AN and PEGMA monomers in a reaction mixture. Thin film composite membranes on the porous PAN support membranes were successfully fabricated from PAN-*r*-PEGMA copolymers solution in ethanol/water (70/30 *v/v* %) mixture by dip coating at RT. The selective layer thickness depends on the concentration of the PAN-*r*-PEGMA copolymer coating solution. The defect free selective layer was attained from 3 wt % PAN-*r*-PEGMA copolymer solution in ethanol/water (7:3 *w/w*) mixture. The gas fluxes were significantly affected by the PEGMA content of the copolymers. We observed that both CO_2_ permeance and CO_2_/N_2_ selectivity enhanced with an increase in PEGMA content. The PAN-*r*-PEGMA67 composite membrane exhibits a high CO_2_ permeance of 1.37 × 10^−1^ m^3^/m^2^·h·bar and an ideal CO_2_/N_2_ selectivity of 65. The CO_2_ gas permeance and the CO_2_/N_2_ selectivity for PAN-*r*-PEGMA67 composite membrane are slightly higher than the values obtained with membranes made in the same way using commercially available PEBAX1657. PAN-*r*-PEGMA67 composite membranes have great potential for use in CO_2_ separation owing to their excellent selectivity, permeability and easy scalability.

## Figures and Tables

**Table 1:** PAN-*r*-PEGMA with various chemical compositions and their molecular weights.

Sample Code	Mol (%)		PEGMA (Da)	PEGMA wt %	M_w_ (Da)	M_w_/M_n_ (PDI)
	AN	PEGMA				
PAN-*r*-PEGMA40	89.3	10.7	300	40	56,052	2.15
PAN-*r*-PEGMA60	79.0	21.0	300	60	53,669	2.39
PAN-*r*-PEGMA64	83.6	16.4	475	64	43,918	1.96
PAN-*r*-PEGMA67	89.9	10.1	950	67	52,397	3.60

**Table 2:** Membrane thickness, CO_2_ gas permeability and selectivity.

Membranes	Selective Layer	$P_{CO_{2}}$	Selectivity (α)		
	Thickness (μm)	(Barrer)	CO_2_/N_2_	CO_2_/CH_4_	CO_2_/H_2_
PAN-*r*-PEGMA40	1.32	2	52	28	1.6
PAN-*r*-PEGMA60	1.35	10	53	25	3.7
PAN-*r*-PEGMA64	1.23	20	60	23	5.9
PAN-*r*-PEGMA67	1.36	69	65	20	9.8
PEBAX1657	1.38	64	51	18	7.7

1 Barrer = 10^−1^ cm^3^ (STP) cm/(cm^2^ s cmHg).
